# Use of 3D Human Liver Organoids to Predict Drug-Induced Phospholipidosis

**DOI:** 10.3390/ijms21082982

**Published:** 2020-04-23

**Authors:** Ji-Young Lee, Hyo-Jeong Han, Sang-Joon Lee, Eun-Ho Cho, Han-Byul Lee, Ju-Hyung Seok, Hee Seon Lim, Woo-Chan Son

**Affiliations:** 1 Department of Medical Science, Asan Medical Institute of Convergence Science and Technology, Asan Medical Center, University of Ulsan College of Medicine, Seoul 05505, Korea; goodbye9068@hanmail.net (J.-Y.L.); hhyoj91@mail.ulsan.ac.kr (H.-J.H.); tkdwns516@gmail.com (S.-J.L.); jhonho0724@gmail.com (E.-H.C.); onestar0620@naver.com (H.-B.L.); sjh4409@gmail.com (J.-H.S.); lims3858@mail.ulsan.ac.kr (H.S.L.); 2 Department of Pathology, University of Ulsan College of Medicine, Asan Medical Center, Seoul 05505, Korea

**Keywords:** 3D human liver organoids, drug-induced phospholipidosis, HepG2

## Abstract

Drug-induced phospholipidosis (PL) is a storage disorder caused by the formation of phospholipid-drug complexes in lysosomes. Because of the diversity of PL between species, human cell-based assays have been used to predict drug-induced PL in humans. We established three-dimensional (3D) human liver organoids as described previously and investigated their liver characteristics through multiple analyses. Drug-induced PL was initiated in these organoids and in monolayer HepG2 cultures, and cellular changes were systemically examined. Organoids that underwent differentiation showed characteristics of hepatocytes rather than HepG2 cells. The organoids also survived under PL-inducing drug conditions for 48 h and maintained a more stable albumin secretion level than the HepG2 cells. More cytoplasmic vacuoles were observed in organoids and HepG2 cells treated with more potent PL-induced drugs, but to a greater extent in organoids than in HepG2 cells. Lysosome-associated membrane protein 2, a marker of lysosome membranes, showed a stronger immunohistochemical signal in the organoids. PL-distinctive lamellar bodies were observed only in amiodarone-treated organoids by transmission electron microscopy. Human liver organoids are thus more sensitive to drug-induced PL and less affected by cytotoxicity than HepG2 cells. Since PL is a chronic condition, these results indicate that organoids better reflect metabolite-mediated hepatotoxicity in vivo and could be a valuable system for evaluating the phospholipidogenic effects of different compounds during drug development.

## 1. Introduction

Drug-induced phospholipidosis (PL) is a phospholipid storage disorder caused by the formation of phospholipid-drug complexes in lysosomes, characterized as lamellar bodies by transmission electron microscopy [1]. Although the molecular mechanisms underlying drug-induced PL are still not fully understood, they are thought to be associated with the formation of an indigestible phospholipid–drug complex, the suppression of phospholipase activity, disrupting membrane fluidity [2], and an imbalance between the production and degradation of phospholipids [3,4]. Drug-induced PL is generally associated with amine-containing cationic amphiphilic drugs (CADs), which are protonated and captured in the lysosome’s acidic environment, causing remarkable tissue accumulation of the drug [5].

The controversy continues over whether PL is a toxic or adaptive response. PL is generally considered to be an adaptive response rather than an adverse effect of a particular drug at the early stages [6]. PL can occur in target organs without organ-related toxicity or physiological consequences [7], however, there are cases in which PL is observed with target organ toxicity [8]. Due to the uncertainty of the toxicological outcome of PL, drug-induced PL discovered in preclinical studies can have a detrimental impact on pharmaceutical development and delay the process. Fatal genetic disorders such as Niemann–Pick disease and Tay–Sachs disease [9,10] associated with PL have been described in humans, as has cell membrane damage leading to cell death. And other hepatic [11,12] cardiac [13,14], renal [15,16] pulmonary [17,18], eye [19], nervous [20], and skin [21] toxicities related to PL are known.

Although drug-induced PL can be detected preclinically using in vivo systems such as animal models [3,22,23,24], these types of studies are expensive, time-consuming, can raise ethical issues, and may not have specific toxicological relevance to humans. It has also been recommended that compounds that can lead to PL in humans should be identified and screened out in the early stages of drug discovery [25]. PL can be observed in any organ in the body, but the lung, liver, kidney, brain, spleen, and other lymphoid tissues are the most commonly involved sites [26]. To identify chemicals that provoke PL in humans, cell-based in vitro assays using various human liver cell types, such as HepG2 cells, HuH7 cells, and human primary hepatocytes have been investigated [27,28,29]. Immortalized cell lines derived from human hepatocellular carcinomas have the advantage of being easy to culture and quick growing. However, they do not represent a normal human liver because they retain the transformed characteristics of cancer cells. By comparison with cancer cell lines, primary human hepatocytes have characteristics that much more closely reflect a normal human liver, and also the individual patient. They are therefore more suitable for a toxicity test, but have the distinct disadvantage of losing viability very quickly in such testing, often within one week. Furthermore, the quantities of viable cells that can be obtained from primary tissues are always limited and hepatocytes themselves are fully differentiated and rarely proliferate in culture.

Organoids are a recently developed three-dimensional (3D) cell culture system for adult organs which enables the in vitro expansion of animal or human primary tissues [30]. Duct cells from an adult human liver form sphere-shape 3D structures in extracellular matrices. These so-called “organoids” can expand and proliferate through passaging in culture, unlike other primary cells, and they adopt hepatic characteristics when induced to differentiate by exposure to hepatocyte growth factors for 10–12 days. Human liver organoids can thus show properties of a normal liver or of the diseased condition of an individual patient. In addition, their unique 3D structures better reflect an in vivo environment compared to conventional two-dimensional (2D) cultures. Organoids are promising candidates for toxicity assessment, and several liver organoid models have been used to evaluate drug-induced hepatotoxicity by in vitro approach [31,32,33]. However, the reported toxicity studies with liver organoids are limited to assess cytotoxicity simply, and multiple applications of assays suitable for certain toxicologic phenomena such as phospholipidosis have not been reported yet.

In our current study, we established 3D human liver organoids in accordance with a method described previously [30]. To assess the feasibility of using adult human liver organoids to predict drug-induced PL, we compared the effects of drug-induced PL in these structures and in the HepG2 cell line. HepG2 is widely used [27,29,34,35] for the in vitro screening for this PL condition and plenty of studies have been reported using this. Cellular in vitro endpoints for PL usually include fluorescent phospholipid accumulation in 2D cultured cells or changes in gene expression [26]. For the more in-depth investigation for PL, we conducted multiple examinations of the cellular changes in both systems to analyze the onset of PL, including cell viability, albumin secretion, morphology, protein immunostaining, ultra-microstructure analysis by transmission electron microscopy, and gene expression analysis. The results indicate that organoids better reflect metabolite-mediated hepatotoxicity in vivo and could be a valuable system for evaluating the phospholipidogenic effects of different compounds during drug development.

## 2. Results

### 2.1. Liver Cell Characterization

**Figure 1:**
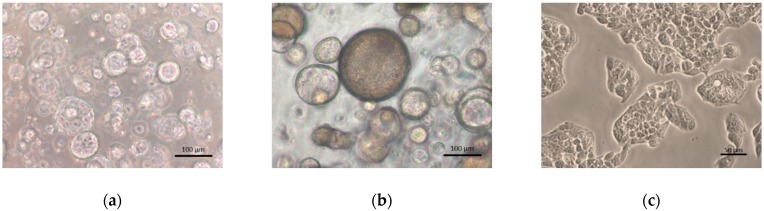
Morphology of cultured hepatocyte systems. (**a**) Three-dimensional human liver organoids on day 21 in expansion medium (EM); (**b**) 3D human liver organoids on day 9 in differentiation medium (DM); (**c**) HepG2 cells grown in monolayer culture. Original magnifications, ×100 (**a**,**b**) and ×200 (**c**). Bar indicates 100 μm (**a,b**) and 50 μm (**c**).

The morphology of the 3D liver organoids and 2D HepG2 cells were compared as shown in Figure 1. Organoids cultured using an expansion medium (EM) appear as transparent spheres that have expanded in size. When organoids undergo differentiation, however, they show a compact and darkened appearance, and each cell adopts a hexagonal shape. HepG2 cells, on the other hand, grow as a monolayer and have a flattened appearance.

#### 2.1.1. Hepatic Marker Expression

**Figure 2:**
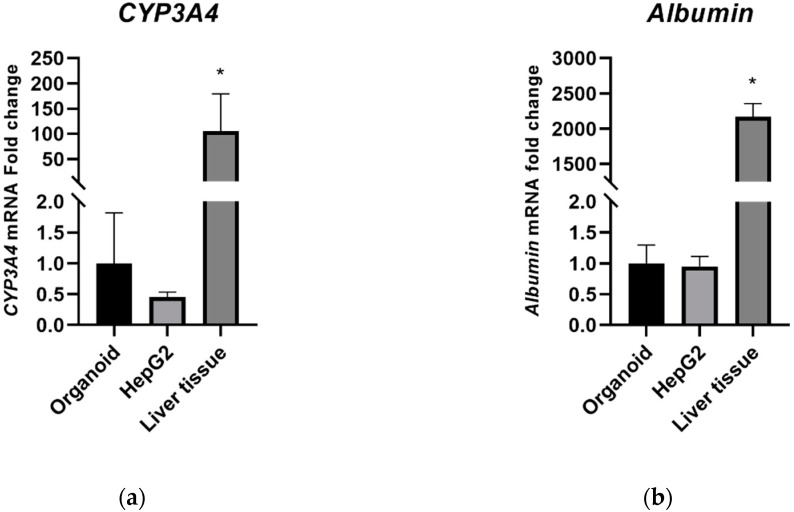
CYP3A4 and albumin mRNA expression in organoid and HepG2 cells. (**a**) 2-dCt values of CYP3A4; (**b**) 2-dCt values of albumin. Data are shown as a mean ± standard error; * *p* < 0.05. The experiments were performed in triplicate, each being repeated at least three times.

*CYP3A4* and *albumin* gene expression was analyzed by quantitative real-time PCR (qPCR). RNA extracted from human liver tissue was used as a positive control. The mRNA expression level of CYP3A4 in organoids was marginally higher than that in HepG2 cells (Figure 2a). The mRNA expression level of albumin was comparable, however, between the organoids and HepG2 cells (Figure 2b). The mRNA expression levels of CYP3A4 and albumin significantly higher in liver tissues than in the organoid and HepG2 culture systems.

#### 2.1.2. Glycogen Storage

**Figure 3:**
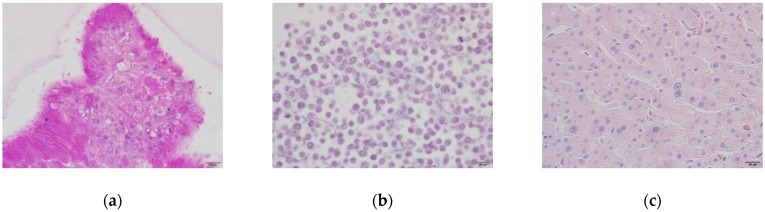
Glycogen accumulation in organoids and HepG2 cells. Polysaccharides or mucosubstances were stained positively with a magenta color. (**a**) DM day 12 organoids; (**b**) HepG2 cells; (**c**) Human liver tissue. Original magnification, ×400. Bar indicates 20 μm.

Glycogen accumulation in human liver organoids, HepG2 cells, and human liver tissue was evaluated by Periodic Acid Schiff (PAS) staining. The organoids showed clear positive glycogen accumulation through the appearance of strong magenta color staining. This staining in HepG2 cells was not as clear as much as organoids. The organoid cells contained polysaccharides or mucosubstances such as glycogen, glycoproteins, and glycolipids in their cytoplasm, as shown in Figure 3.

#### 2.1.3. Hepatic Protein Expression

**Figure 4:**
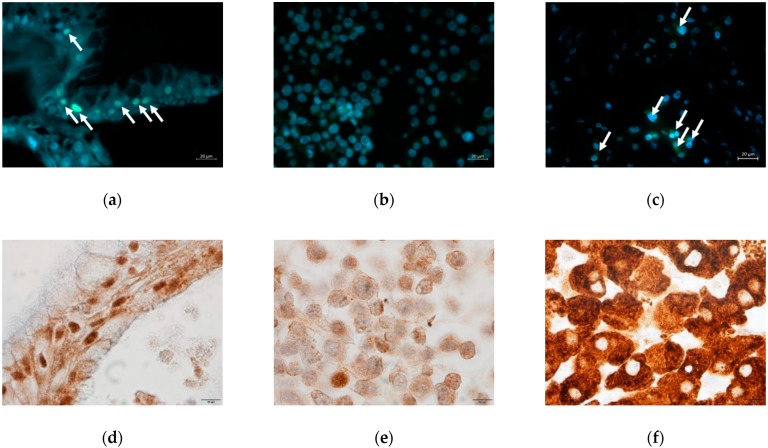

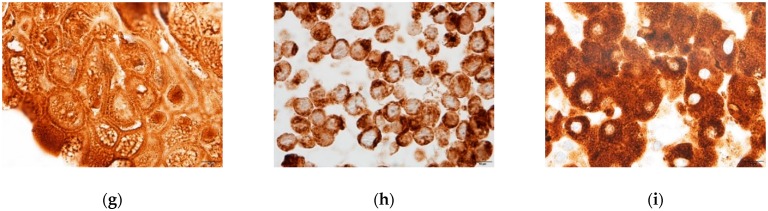
Immunostaining of HNF4α (**a**–**c**), CYP3A4 (**d**–**f**), and CYP1A2 (**g**–**i**). (**a,d,g**) DM day 12 organoids; (**b,e,h**) HepG2 cells; (**c,f,i**) Human liver tissue. Green florescent signals indicated positive staining for HNF4α (white arrows). Brown color signals indicated positive staining for CYP3A4 or CYP1A2. Original magnifications, ×400 (**a**–**c**) and ×1000 (**d**–**i**). Bar indicates 20 μm (**a**–**c**), and 10 μm (**d**–**i**).

The hepatic protein HNF4α was detectable by immunofluorescence in both culture systems. As shown in Figure 4a–c, human liver organoids showed positive nuclear staining (green) for this protein which was stronger than that in the HepG2 cells. Non-specific positive staining was seen in the cytoplasm of HepG2 cells.

CYP3A4 and CYP1A2 expression were visualized by immunohistochemistry in both culture systems. As shown in Figure 4d–f, CYP3A4 expression in human liver organoids was more distinct than in HepG2 cells. CYP1A2 expression was clearly positive in both hepatocyte cultures (Figure 4g–i).

### 2.2. Cell Viability Changes

**Figure 5:**
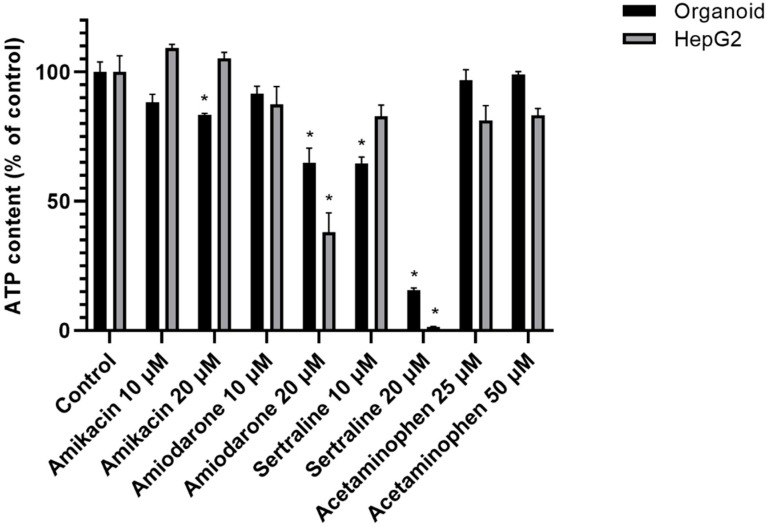
Cell viability changes in organoids and HepG2 cells. Human liver organoids and HepG2 cells were incubated with the indicated drugs (amikacin, amiodarone, sertraline, and acetaminophen) for 48 h. A non-treatment group was used as a control (Con). Cell viability was assessed via the detection of ATP luminescence. Data are shown as a mean ± standard error; * *p* < 0.05. The experiments were performed in triplicate, each being repeated at least three times.

Three-dimensional human liver organoids were found to survive better when exposed to a high dose of a PL-inducing drug than HepG2 cells after 48 h of incubation. The viability of organoids and HepG2 cells changed drastically in every PL drug group. Both 2D and 3D cultured hepatocytes were killed by exposure to sertraline, a strong PL-inducing drug. Monolayers of HepG2 cells showed obvious cell death at a 20 μM dose of amiodarone and sertraline, whilst the viability of the organoids was higher at these drug concentrations. Acetaminophen was used at 25 μM or 50 μM levels and caused no significant cell viability changes in either culture system. These cell viability data are shown in Figure 5.

### 2.3. Albumin Secretion Content

**Figure 6:**
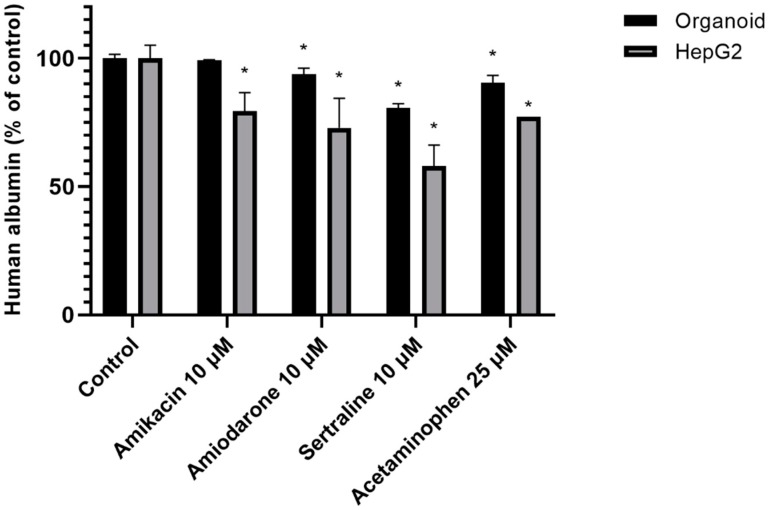
Secreted albumin levels from organoids and HepG2 cells. Organoids and HepG2 cells were incubated with the indicated drugs (10 μM amikacin, amiodarone, sertraline, and 25 μM acetaminophen) for 48 h. A non-treatment group was used as a control. Data are shown as a mean ± standard error; * *p* < 0.05. The experiments were performed in duplicate, each being repeated at least three times.

The albumin secretion level from HepG2 cells decreased more sharply than in liver organoids in the presence of a PL-inducing drug (depending on the intensity of its PL-inducing potential). Both hepatocyte culture systems were incubated with 10 μM PL-inducing drugs or 25 μM acetaminophen as a negative control for 48 h. The organoids maintained a more stable albumin secretion capacity than HepG2 cells as shown in Figure 6.

### 2.4. Evaluation of Drug-Induced Phospholipidosis

#### 2.4.1. Morphological Changes

**Figure 7:**
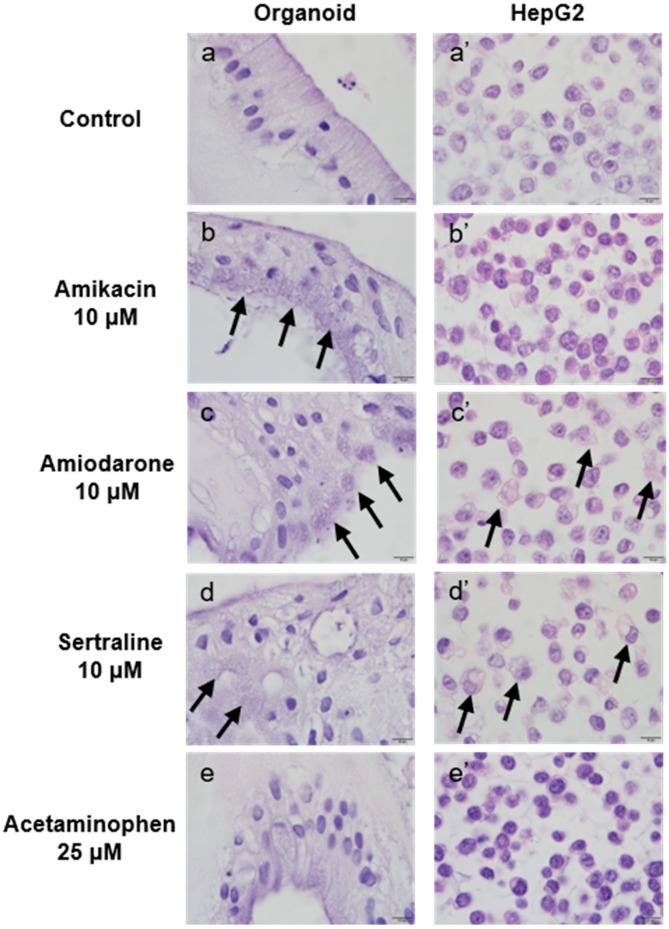
Microscopic evaluation of morphological changes in organoids and HepG2 cells following incubation with the indicated agents. Organoids (**a**–**e**) and HepG2 cells (**a’**–**e’**) were treated with phospholipidosis (PL)-inducing drugs or untreated for 48 h as follows: Control (untreated) (**a**, **a**’), 10 μM amikacin (**b**, **b**’), 10 μM amiodarone (**c**, **c**’), 10 μM sertraline (**d**, **d**’) and 25 μM acetaminophen (**e**, **e**’). Arrows indicate PL-induced cytoplasmic vacuolation. Original magnification, ×1000. Bar indicates 10 μm.

To assess morphological changes in the organoid and HepG2 cell culture systems resulting from drug-induced PL, the cells were treated with 10 μM of the indicated drugs or 25 μM acetaminophen for 48 h, embedded in cell blocks, and stained with H&E. As shown in Figure 7, drugs with a higher potency for PL induction caused higher cytoplasmic vacuole production in both culture systems.

#### 2.4.2. Comparison of LAMP-2 Expression

**Figure 8:**
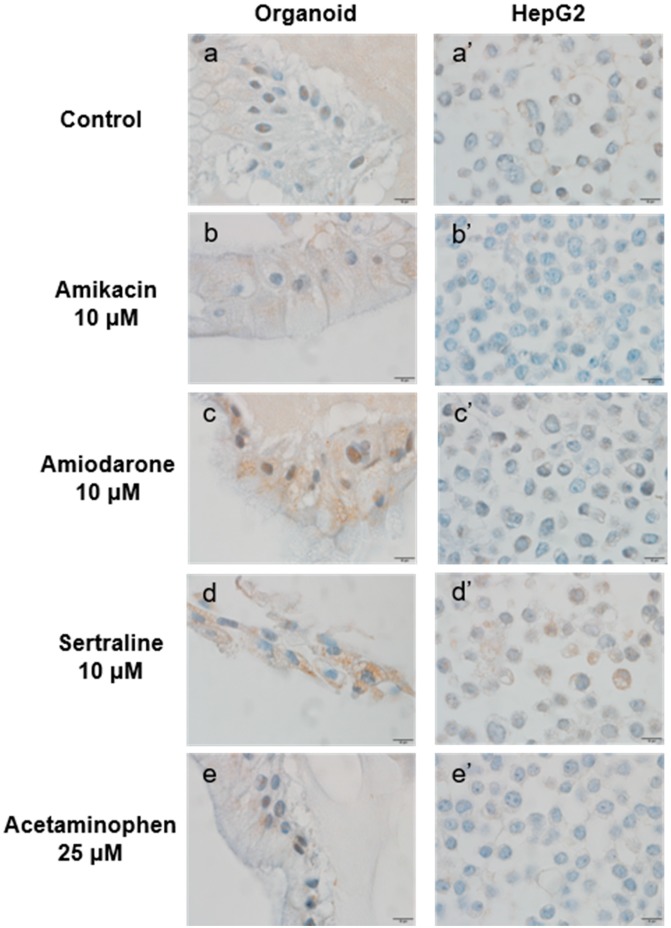
LAMP-2 expression in organoids and HepG2 cells following incubation with the indicated agents. Organoids (**a**–**e**) and HepG2 cells (**a’**–**e’**) were treated with the indicated PL-inducing drugs or untreated for 48 h as follows: Control (untreated) (**a**, **a**’), 10 μM amikacin (**b**, **b**’), 10 μM amiodarone (**c**, **c**’), 10 μM sertraline (**d**, **d**’), and 25 μM acetaminophen (**e**, **e**’). Brown signals indicate positive staining for LAMP-2. Original magnification, ×1000. Bar indicates 10 μm.

Phospholipid accumulation was detected by immunohistochemical staining for LAMP-2 (Figure 8). The cells were again incubated with 10 μM of a PL-inducing drug (amikacin, amiodarone, or sertraline) or 25 μM acetaminophen for 48 h. The organoid cells had more apparent LAMP-2 positive staining in the cytoplasm compared with the HepG2 cells under the same conditions. Both kinds of cells in the sertraline-treated group showed the most strongly positive cytoplasmic staining.

#### 2.4.3. Confirmation of Drug-Induced Phospholipidosis

**Figure 9:**
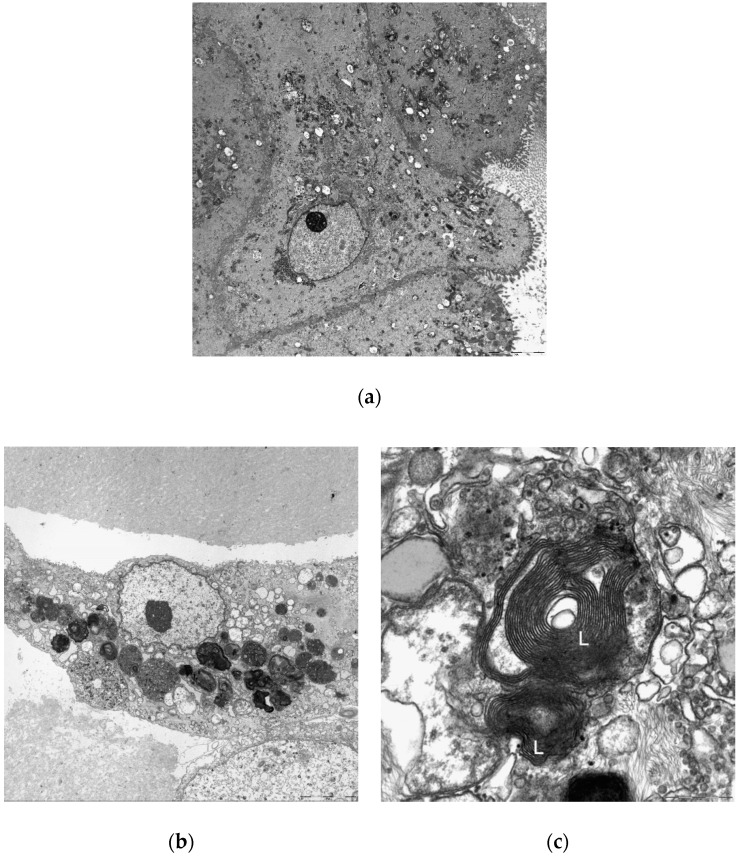
Transmission electron microscopic evaluation of the organoids, which had been differentiated for 12 days and then treated as follows: (**a**) Control (untreated); (**b, c**) 10 μM amiodarone incubation for 48 h. Lamellar bodies are indicated by ‘L’. Original magnifications, ×3000 (**a, b**) and × 40,000 (**c**).

Transmission electron microscopy was used to confirm drug-induced phospholipidosis in the liver organoids (Figure 9). The organoids treated with 10 μM amiodarone for 48 h showed a clear production of lamellar bodies, which were not evident in the control group. Cell to cell junctions within the organoids and bile canaliculi were also observed.

#### 2.4.4. Gene Expression Changes under Conditions of Drug-Induced Phospholipidosis

**Figure 10:**
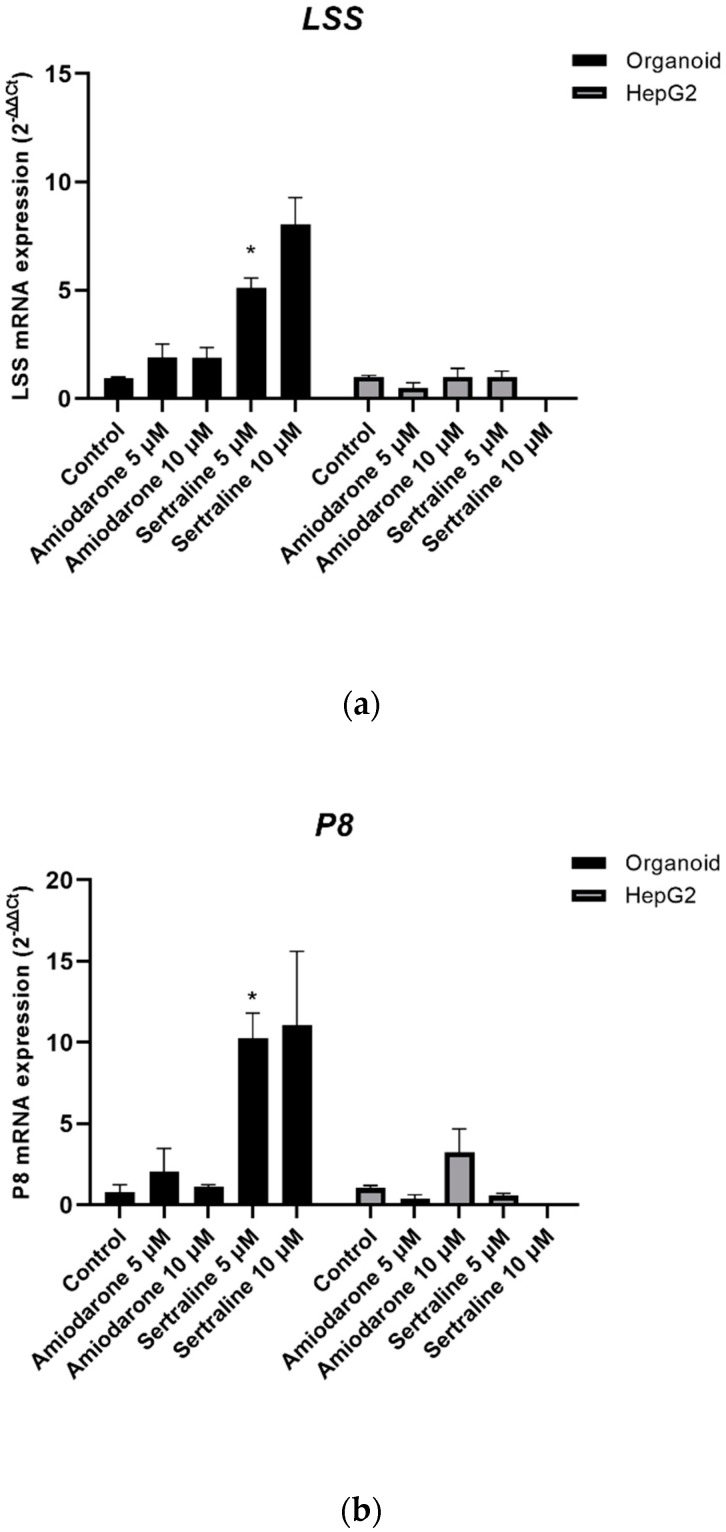
Fold changes in the gene markers of phospholipidosis in organoids and HepG2 cells. Fold changes were measured by the 2^−ΔΔ^*^C^*^t^ values of gene markers (**a**) LSS and (**b**) P8. mRNA fold changes are shown as a mean ± standard error; * *p* < 0.05. The experiments were performed in triplicate, each being repeated at least three times.

To evaluate the severity of drug-induced PL, the fold-change values for specific gene markers of this phenomenon were measured by qPCR. The cells in both culture systems were treated with amiodarone (5 μM, 10 μM) or sertraline (5 μM, 10 μM) for 48 h. HepG2 cells were found to be extremely sensitive to 10 μM sertraline toxicity and RNA extraction was therefore not possible under this condition. As indicated in Figure 10, the LSS and P8 mRNA expression levels of organoids showed an upregulation, as previously reported [36].

## 3. Discussion

PL induction by certain drugs is a potential problem for human pharmaceutical development. From a regulatory perspective, drug-induced PL also has several toxicological implications. When this phenomenon arises in lysosomes upon exposure to a drug concentration above 10 μM, it can result in a disruption to cellular membrane integrity and blockage of phospholipid degradation [3]. If this condition then continues throughout the prolonged administration of a particular drug, it may impact cellular function or even cause cell death [7]. Moreover, many other side effects are reported to be associated with drug-induced PL, such as cardiotoxicity (such as QT prolongation), myopathy, hepatotoxicity, nephrotoxicity, and pulmonary dysfunction [37].

Although PL is a frequent histological finding in exploratory preclinical in vivo studies, it is not certain that its occurrence in animals will be toxicologically relevant to humans. Differences in the phospholipase enzyme between humans and other animal species may be a plausible explanation for the poor predictive capacity of animal studies for this disorder [38]. Hence, it is desirable to identify drugs in their early developmental stages that have the potential to induce PL in humans [25]. Cell-based in vitro assays using various types of cells of human liver origin have been utilized in the past to screen for chemicals that provoke PL in humans. Significantly, we applied a robust 3D human liver organoid system to this purpose. We here describe the various examination of cellular alterations that occur during the onset of drug-induced PL in liver organoids, including viability changes, albumin secretion levels, morphological changes, and protein expression changes determined by immunostaining. We also confirmed that phospholipidosis is induced in organoids by detecting pathognomonic microstructural alterations through transmission electron microscopy. We further conducted the expression analysis of known PL marker genes.

Our detailed analyses of human liver organoids in this present study were conducted in parallel with the established HepG2 liver cell line that has been typically used for this type of screening [28,29,34] in the past. Organoid cells that undergo differentiation show characteristics that are far more similar to primary hepatocytes than HepG2 immortalized liver carcinoma cells. The mRNA expression levels of CYP3A4 and albumin in both organoids and HepG2 cells were marginal compared with normal human liver tissue. However, the nuclear expression of HNF4α, a hepatic transcription factor, was far stronger in organoids, as determined by immunofluorescence. CYP3A4 and CYP1A2 expression were detectable in both cell cultures by immunohistochemistry. In PAS staining experiments, however, the organoids showed clear glycogen accumulation, whereas the HepG2 cells rarely stained positively.

The impacts of drug-induced PL on our 3D organoid and 2D hepatocyte culture systems were evaluated. As shown in Figure 5, the 3D human liver organoids survived in the presence of a high dose PL-inducing drug that was toxic to HepG2 cells at that same concentration over a 48-h incubation. The viability of HepG2 cells changed drastically in every PL drug group, and the number of HepG2 cells in the amikacin group actually showed a slight increase over the vehicle control group. Both 2D and 3D cultured hepatocytes were killed at a 20 μM dose of amiodarone and sertraline, but the viability of organoids was higher than that of HepG2 cells. This survival change in HepG2 cells seems to be caused by the monolayer growth conditions that lead to a much wider exposure area to the drug. Additionally, since cells in vivo interact by forming close cellular junctions with surrounding cells, the lesser viability changes in organoids due to greater drug resistance are more likely to reflect an *in* vivo environment. It is significant in this regard also that the 3D liver organoids showed a more stable albumin secretion level than the HepG2 cell monolayers.

In our morphological evaluations of PL, the more potent PL-inducing drugs led to the production of more cytoplasmic microvesicular vacuoles in both the organoids and HepG2 cells. Although these foamy changes are presumed to be a result of drug-induced PL, these vacuolated entities caused by this condition resemble lipid accumulation or artifacts that can arise during the processing of a tissue slide for light microscopy. To clearly delineate that they were indeed the result of PL induction, we conducted an immunohistochemical analysis of LAMP-2, a specific marker of lysosome membranes that would clearly distinguish PL from lipid droplets. The expression of LAMP-2 was more evident in organoids than in HepG2 cells under the same PL-inducing conditions. To further confirm drug-induced PL in the organoids, differentiated organoids cultured with/without 10 μM amiodarone for 48 h were examined under a transmission electron microscope. PL-distinctive lamellar bodies were observed only in the amiodarone-treated organoids. The observation of lamellar bodies in organoids following PL-inducing drug treatment may suggest that organoids are relatively sensitive to phospholipidosis. In addition, the ultra-microstructures of the organoids were found to include cell to cell junctions and bile canaliculi. The LSS and P8 mRNA levels were also found to be up-regulated in organoids as described previously [35,36]. Interestingly, the mRNA expression changes related to PL induction that were previously described [36] were not reproduced in HepG2 cells in our current experiments.

Our present study findings further revealed that 3D cultured human liver organoids are more sensitive to drug-induced PL and less affected by cytotoxicity than HepG2 cell monolayers. Since PL is a chronic cellular alteration, rather than cytotoxic acute toxicity, these results support the notion that 3D-structured liver organoids better reflect metabolite-mediated hepatotoxicity in vivo than a monolayer culture [39,40]. It has been reported in this regard that the detectable toxic concentration (25 μM) of tetracycline in gel entrapped 3D cultured rat hepatocytes treated for 96 h is very close to the actual toxic serum concentration in rats (27 μM) [41]. In contrast, and somewhat understandably, the HepG2 cells in our current experiments showed a clear decrease of viability and loss of liver function that does not correspond to the *in* vivo environment. Two-dimensional cultured HepG2 cells showed changes resulting from a greater drug dose under 2D in vitro culture conditions. The higher sensitivity of organoids to drug-induced PL further suggests that these structures are a far better match for the in vivo situation. Although we used organoids from a single patient in this study, if we could obtain the liver tissues and establish normal liver organoids from more people, the organoids will be able to be applied as a precise toxicity screening system that responds to PL-inducer according to the individual patient’s characteristics.

It is difficult to predict drug-induced hepatotoxicity, especially chronic hepatotoxicity such as PL, using in vitro systems because of the intrinsic limitations of cells grown outside of the body [42]. Organoids themselves are composed of a single type of cell from the liver so they are also an imperfect system for predicting likely drug responses in vivo. Moreover, the number of cells and the growth rate of each organoid are difficult to control. Co-culturing with other types of cells, such as immune cells [43] or endothelial cells [44], can make the microtissue more similar to in vivo environment, and many other studies are ongoing in this regard. The development of more reliable and appropriate cell systems for toxicity screening is thus still necessary for the early prediction of drug toxicity in vivo.

## 4. Materials and Methods

### 4.1. Chemicals

The PL-inducing agents and other compounds tested in the assays, including amikacin, amiodarone hydrochloride, sertraline hydrochloride, and acetaminophen, were purchased from Sigma-Aldrich (St. Louis, MO, USA). Information about each of these drugs can be found in prior reports [7,35]. Amikacin, amiodarone, and sertraline are weak, moderate and strong PL-inducing drugs, respectively. Acetaminophen was used as a negative control drug for the induction of PL. All other compounds and reagents used in the analyses were obtained at an analytical grade from common commercial sources.

### 4.2. Cell Culture

#### 4.2.1. Human Liver Organoids

Human liver tissue (0.5–1 cm^3^) from a single patient was obtained from the hepatectomy performed at Asan Medical Center, Seoul, with informed consent given by the patient and approval by the Institutional Review Board of Asan Medical Center (Approval date: 20 July 2017, approval no. S2017-0969-0003). After surgical excision, the tissue was kept at 4 °C in Hanks’ Balanced Salt Solution (HBSS) until processing. Human liver organoids were generated in accordance with the protocols of Broutier et al [30]. Briefly, the tissue was chopped with sterile scissors and digested by collagenase D (Roche) and DNase I (Sigma) in sterile Earle’s Balanced Salt Solution (EBSS) medium (Thermo Fisher Scientific, Waltham, MA, USA). The isolated duct cells were then mixed with Matrigel (BD Biosciences, San Jose, CA, USA) or reduced growth factor BME 2 (Basement Membrane Extract, Type 2, Pathclear) (Amsbio, Abingdon, UK), and 5000–10,000 cells were seeded per well in a 24-well plate. After the matrigel or BME had been solidified, 500 μL of culture medium was added per well. The expansion medium (EM) for the organoids was based on advanced DMEM/F12 (Invitrogen, Waltham, MA, USA) and various supplements were included. For the first 3 days, the medium was supplemented with 25 ng/mL recombinant human noggin (Peprotech, Rocky Hill, NJ, USA), 100 ng/mL Wnt3a (Peprotech), and 10 µM Y27632 (Sigma Aldrich) to isolate the duct cells. The medium was then replaced with EM without noggin, Wnt3a, or Y27632. After a single duct cell had budded to an organoid, passaging was performed at a 1:2–1:4 split ratio once every 5–7 days. For normalization of the number of cells in each well, the organoids were dissociated into single cells using TrypleLE (Gibco, Waltham, MA, USA) and seeded with the same number per well. The cells were then cultured at 37 °C in a humidified atmosphere containing 5% CO_2_. 

To differentiate organoids derived from hepatic progenitor cells in hepatocytes, the seeded organoids were maintained in culture for 5 days in EM supplemented with BMP7 (25 ng/mL) which was then replaced with differentiation medium (DM). The DM was advanced DMEM/F12 medium supplemented with 1% N2 and 1% B27 and containing 50 ng/mL recombinant human EGF, 10 nM gastrin (Sigma), 25 ng/mL recombinant human HGF, 100 ng/mL recombinant human FGF19 (R and D, Minneapolis, MN, USA), 500 nM A83-01, (10 µM DAPT (Sigma)), 25 ng/mL recombinant human BMP7 (Peprotech) and 30 µM dexamethasone (Sigma). Fresh DM was added every 2–3 days for a period of 10–12 days.

#### 4.2.2. HepG2 Cells

The human hepatocellular carcinoma cell line HepG2 was kindly provided by Dr. In Kyong Shim from the Asan Institute for Life Sciences (Seoul, Republic of Korea). These cells were cultured in DMEM supplemented with 10% fetal bovine serum (FBS), 100 U/mL penicillin and 100 g/mL streptomycin in 175T culture flasks. The cultures were maintained in a humidified atmosphere with 5% CO_2_ at 37 °C and subcultured every 3 or 4 days using 0.05% trypsin/EDTA in PBS.

### 4.3. Treatment of Cells

The 2D and 3D cultured cells were incubated in media supplemented with the chemical compound of interest for 48 h. For subculturing, dissociated human liver organoid cells were seeded at 10,000 cells per well in 24 well plates. Differentiation was induced for 10 days when the diameter of the organoids did not exceed 200 μm to avoid apoptosis. On day 10 of differentiation, the compounds being tested (5 or 10 μM for amikacin, amiodarone, and sertraline; 25 or 50 μM for acetaminophen) were added to the DM in separate experiments. HepG2 cells were seeded onto the culture plates at a suitable density for each assay. After the cells had adhered to the plates following an overnight incubation, the culture media was replaced and supplemented with each chemical.

### 4.4. RT-qPCR

As hepatic functional markers, the mRNA expression of CYP3A4 and albumin in the organoids and HepG2 cells were compared by quantitative real-time PCR (qPCR). Cells were harvested by Trizol and stored at −80 °C until RNA extraction using chloroform as described previously [45]. The concentration and purity of the total RNA preparations were determined by measuring the absorbance at 260 and 280 nm with a NanoDrop (Thermo Fischer Scientific). To synthesize cDNA, reverse transcription (RT) was performed using the RevertAid First Strand cDNA Synthesis kit (Thermo Fisher Scientific). Briefly, 1000 ng of total RNA was reacted in a 20 μL mixture including oligo-dT oligonucleotide primer, RevertAid reverse transcriptase, and RiboLock RNase inhibitor. For CYP3A4, the forward oligo was 5′-CTTCATCCAATGGACTGCATAAAT-3′, and the reverse oligo was 5′-TCCCAAGTATAACACTCTACACAGACAA-3′. For albumin, the forward oligo was 5′-ATGCCCCGGAACTCCTTTTC-3′, and the reverse oligo was 5′-CAACAGGCAGGCAGCTTTAT-3′. qPCR was performed using the ABI PRISM 7900HT Sequence Detection System (Thermo Fischer Scientific) with the following amplification protocol: 2 min at 50 °C and 2 min at 95 °C, followed by 40 cycles of denaturation for 15 s at 95 °C, and annealing and elongation for 1 min at 60 °C in a final volume of 10 μL. The relative gene expression levels in the two culture systems were normalized to that of glyceraldehyde-3-phosphate dehydrogenase (GAPDH) and calculated using the comparative Ct method.

To confirm the drug-induced PL in mRNA gene expression, the fold changes of several gene markers (*LSS* and *P8*) following drug-induced PL were measured by qPCR. *LSS* is associated with lipid metabolism/cholesterol biosynthesis and *P8* is concerned with cell cycle, proliferation, and death. Those genes were selected according to the previous report about a toxicogenomic approach to drug-induced PL analysis [36]. Amiodarone (5, 10 μM) and sertraline (5, 10 μM) were used to treat the cells for 48 h. The cells were then collected, and their RNA was extracted with Trizol. After checking the concentration and purity of the total RNA, cDNA synthesis and qPCR were conducted as indicated above. Relative gene expression levels were normalized to a housekeeping gene, and the results were calculated by 2^−∆∆Ct^ and compared with a vehicle control.

### 4.5. PAS Staining

Non-drug treated HepG2 cells and differentiated human organoids were fixed with 10% neutral buffered formalin for 30 min. After washing the cells twice with phosphate-buffered saline (PBS), they were collected into a 1.5 mL tube and gently centrifuged to preserve the cellular structure. The resulting cell pellets were then embedded in Histogel. The solidified gel blocks containing the cells were transferred to tissue cassettes and tissue paraffin-embedded blocks were created using a standard method. Formalin-fixed human liver tissue was made to a paraffin-embedded block. To determine the glycogen storage levels in organoids HepG2, and human liver tissue, 3 μm-sectioned slides were treated with Periodic Acid Schiff (PAS) which positively stains glycogen, neutral mucins, some epithelial mucins, and the basement membranes of fungal walls (magenta color).

### 4.6. Immunostaining (Immunofluorescence and Immunohistochemistry)

The protein encoded by the *HNF4A* gene is a nuclear transcription factor that binds DNA and regulates several hepatic genes. Immunofluorescence was used to identify whether this liver-related protein was expressed in the organoids or HepG2 cultures. Briefly, 3 μm-sectioned paraffin-embedded cell slides were deparaffinized and antigen retrieval was performed by exposure to citrate buffer at 95 °C for 15 min. A primary anti-HNF4α antibody (Abcam, Cambridge, UK) was incubated with the slide for 2 h at a 1 μg/mL concentration at room temperature. Alexa Fluor^TM^ 488 goat anti-mouse IgG (Invitrogen) was then incubated with the sample at a 1:1000 dilution for 1 h at room temperature. DAPI was used for nuclear counterstaining. Images were acquired under a fluorescent microscope (Observer.Z1, ZEISS, Oberkochen, Germany).

For identifying CYP3A4 and CYP1A2 expression, Immunohistochemistry was conducted. Paraffin-embedded cell blocks were sectioned and stained automatically using a Discovery XT Autostainer (Ventana Medical Systems, Oro Valley, AZ, USA). All reagents used for this automated immunohistochemistry were sourced from Ventana Medical Systems. The primary anti-CYP3A4 antibody (Abcam) and anti-CYP1A2 antibody (Abcam) were used at 1:500 dilution for 36 min. The target antigen was visualized via a DAB reaction. Images were captured under a light microscope (BX53, Olympus, Tokyo, Japan).

### 4.7. Histological Examination

To investigate the morphological changes caused by the PL-inducing drug treatments, the cells were incubated with 10 μM amikacin, amiodarone, and sertraline and 25 μM acetaminophen for 48 h. Cell block slides (3 μm-sections) were then prepared in the same way described formerly. After hematoxylin and eosin staining was conducted, the morphological features of the HepG2 cells and adult liver organoids were thereby evaluated.

### 4.8. Detection of Lysosome-Associated Membrane Protein 2 (LAMP-2)

To differentiate PL from other changes in the liver, immunostaining for LAMP-2 was conducted as previously described [46]. LAMP-2 is a promising marker for PL which has been characterized by immunohistochemistry in rat tissues treated with PL-inducing drugs [47], or cells treated with chloroquine [48]. Briefly, both hepatocyte culture systems were incubated with 10 μM amikacin, amiodarone, sertraline and 25 μM acetaminophen for 48 h. Paraffin-embedded cell blocks were subsequently made, and each was sectioned and stained automatically using a Discovery XT Autostainer (Ventana Medical Systems). A primary anti-LAMP-2 antibody (Invitrogen) was used at a 1:200 dilution for 40 min. The target antigen was visualized via a DAB reaction as described previously.

### 4.9. Cell Viability Assay

Organoids and HepG2 cells were incubated with 10 and 20 μM of amikacin, amiodarone, and sertraline. Acetaminophen was added to both cell culture systems at 25 and 50 μM concentrations. The ATP content in the cells was determined using the ATPlite luminescence ATP detection assay system (Perkin Elmer, Waltham, MA, USA). Briefly, cells were treated with 100 μL of cell lysis solution for 5 min followed by the addition of substrate reagent for 5 min at room temperature. After adaptation in the dark for 10 min, measurements were taken with a luminometer (VICTOR X2, Perkin Elmer). Data are presented as relative percentages to the control value, without drug exposure.

### 4.10. Albumin Content Measurement

Cultured media from the HepG2 cells and human liver organoids were collected before and at 48 h after chemical treatment. The cells were incubated with 10 μM amikacin, amiodarone, sertraline, and 25 μM acetaminophen. The albumin content secreted into the culture medium was assayed using a human albumin ELISA kit (Abcam) in accordance with the manufacturer’s instructions. Data were reported as % value from the control that the cells no PL-inducer was treated.

### 4.11. Identification of Lamellar Bodies

The liver organoids were treated with 10 μM amiodarone for 48 h during differentiation days 10–12. Differentiated organoids with no treatment were used as controls. The cells were then fixed in 2.5% glutaraldehyde solution and stored at 4 °C for embedding and ultrathin sectioning. The fixed samples were then treated with a 1:1 (*v*/*v*) mixture of quetol and dry ethanol for 30 min, followed by two treatments in quetol for 30 and 120 min, respectively. The samples were next mounted on the tips of Beem capsules, which were dried overnight in an oven at 74 °C. The dried samples were sectioned on a microtome and stained with uranyl acetate for 20 min. The samples were counterstained with palladium for a further 4 min, and specimens were viewed under a transmission electron microscope (JEM-1200EX, JEOL, Tokyo, Japan).

### 4.12. Statistical Analysis

All data values are shown as a mean ± SE. Statistical significance was determined by one-way ANOVA test in combination with Student’s t-test with GraphPad Prism 6 (GraphPad Software Inc., La Jolla, CA, USA). *p*-values of less than 0.05 were considered statistically significant. 

## 5. Conclusions

Our findings indicate the feasibility of organoids to predict and screen out the phospholipidosis. The drug-induced PL observed in humans can be successfully reproduced in primary human hepatocytes in 3D cultures and not just in traditional monolayer cultures. Unsurprisingly, human 3D liver organoids better reflect the properties of the primary human liver than hepatocellular carcinoma cell monolayer cultures. The unique cell structure of organoids may make them more resistant to drug-induced toxicity. Three-dimensional human liver organoids thus appear to be a useful and relevant system for evaluating the phospholipidogenic effects of different compounds. As a cell-based approach, 3D human liver organoids can, therefore, be used early in the drug development process to identify chemical agents with the potential to induce PL.

## Abbreviations

3D	three-dimensional
2D	two-dimensional
PL	phospholipidosis
HBSS	Hanks’ Balanced Salt Solution
EBSS	Earle’s Balanced Salt Solution
BME 2	Basement Membrane Extract
EM	expansion medium
DM	differentiation medium
FBS	fetal bovine serum
PBS	phosphate-buffered saline
LAMP-2	lysosome-associated membrane protein 2
qPCR	quantitative real-time polymerase chain reaction
RT	reverse transcription
GAPDH	glyceraldehyde-3-phosphate dehydrogenase
PAS	Periodic Acid Schiff
